# Visible Light Activation for Fungal Biofilm Inhibition: Combining Antimicrobial Photodynamic Therapy with Singlet Oxygen and Iodine Generation against *Candida albicans* and *Pichia kudriavzevii*

**DOI:** 10.1021/acsomega.5c08078

**Published:** 2026-04-13

**Authors:** Gabriela de Souza Calvi, Marilia Toledo Braga, Giulia Nicolle Jácome Cartaxo, Pavel Kubát, Vojtěch Liška, Jiří Mosinger, Maricilia Silva Costa

**Affiliations:** † Instituto de Pesquisa & Desenvolvimento—IP&D, 67655 Universidade do Vale do Paraíba—UNIVAP, Av. Shishima Hifumi, 2911, CEP, 12244-000, São José dos Campos, SP, Brazil; ‡ J. Heyrovský Institute of Physical Chemistry, v.v.i., Academy of Sciences of the Czech Republic, Dolejškova 3, 182 23 Prague, Czech Republic; § Department of Inorganic Chemistry, Faculty of Science, 112302 Charles University, Hlavova 2030, 12843 Prague, Czech Republic

## Abstract

Biofilms formed by *Candida albicans* and the highly resistant *Pichia kudriavzevii* are critical virulence factors because of their resistance to conventional antifungals. This study explored antimicrobial photodynamic therapy (aPDT) using sulfonated polystyrene nanoparticles with an encapsulated tetraphenylporphyrin photosensitizer (TPP-NPs), synergistically enhanced by potassium iodide (KI), to combat biofilms of these yeasts. TPP-NPs irradiated by visible light generate antimicrobial singlet oxygen (O_2_(^1^Δ_g_)), which oxidizes KI to form another reactive species (I_2_/I_3_^–^), augmenting total antimicrobial effects. The usage of TPP-NPs led to reduced cell proliferation and biofilm viability in both species, with KI significantly enhancing efficacy and enabling lower TPP-NP doses. *P. kudriavzevii* biofilms were more susceptible (70–80% inhibition, up to 95% with KI) than *C. albicans* biofilms (30–40% inhibition), a crucial finding for drug-resistant *P. kudriavzevii*. This is the first demonstration that aPDT using TPP-NPs effectively reduces both biofilm formation and viability, especially against resistant *P. kudriavzevii*, highlighting its potential as a biocompatible alternative therapy for biofilm-associated infections.

## Graphical abstract

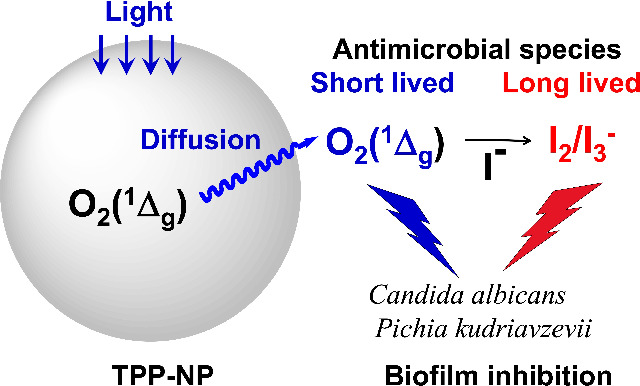

Invasive candidiasis is the most common fungal infection in hospitals worldwide. This leads to several complications for critical care patients in intensive care units in numerous countries every year.^1^ The annual incidence of invasive candidiasis is estimated at approximately 750,000 cases, and 40–55% of cases are lethal infections.^2,3^ Among the most affected individuals are premature infants and elderly patients.^2^ Approximately 40–50% of these cases are caused by *Candida albicans*;^4,5^ however, invasive infections caused by non-*albicans Candida* species have been increasing.^6^ In addition to *Candida albicans,* fungal infections are caused mainly by *Candida glabrata*, *Candida tropicalis*, *Candida parapsilosis* and *Pichia kudriavzevii* (in the past known as *Candida krusei*).^5^ The SENTRY program, which analyzed 20,788 invasive *Candida* isolates from 135 medical centers in 39 countries, reported a reduction in the prevalence of *Candida albicans* from 57.4% (1997–2001) to 46.4% (2015–2016). However, an increase in the prevalence of *Candida glabrata*, *Candida parapsilosis*, *Candida tropicalis*, *Pichia kudriavzevii* (*Candida krusei*), and other less frequently encountered *Candida* species was observed during the same period.^7^ In this context, research on the development of alternative antifungal therapies is imperative.

Many species of yeast are able to produce biofilms, structures composed of adherent cells embedded in an extracellular matrix, which, in comparison with cells in planktonic form, enables greater resistance and virulence.^8–10^ The development of biofilms can cause recalcitrant infections and interfere with the host’s immune response, increasing the probability of acquiring other opportunistic infections.^11^ *Candida albicans* forms biofilms with a complex architecture containing yeast and pseudohyphal and hyphal cells, with significant cell dispersal occurring in the final phase of biofilm development.^12,13^ Additionally, *Candida albicans* filaments serve as “scaffolds” for other species of microorganisms, such as bacteria or fungi, including other *Candida* species, favoring the development of mixed infections.^14–16^ Although less prevalent, *Pichia kudriavzevii* can also produce biofilms in different surfaces, such as polyethylene, polyvinyl chloride, and glass, material present in numerous medical devices,^17^ with significant biomass, metabolic activity and filamentous forms.^18–20^

Biofilm formation by *Candida albicans* and *Pichia kudriavzevii* is a very important condition to the antifungal resistance mechanism, representing a challenge in the public health in the world, by prevent the penetration of antifungal treatment. Biofilms are composed of fungal communities encased in an extracellular matrix (including β-1,3-glucans and extracellular DNA) and represent physical barriers to impede drug penetration, while reduced metabolic activity and overexpression of efflux pumps in biofilm cells further enhance resistance.^10^ As an example, β-1,3 glucan expression in *Candida* biofilms reduces the permeability of traditional antifungals agents, such as amphotericin B and fluconazole. This reduction may be related to the increase in osmotic resistance of the cell wall and the blocking of action sites.^21,22^ Apparently, at each stage of *Candida* biofilms, a predominant resistance mechanism exists. In the earliest stages, efflux pumps exhibit a transient increase during cell adhesion. In mature biofilms, presence of beta-glucans in matrix reduces the permeability and sequesters conventional antifungal agents. Furthermore, in the dispersion phase, the cells exhibit a more persistent phenotypic version.^23^ Nevertheless, *Pichia kudriavzevii* is an emergent pathogen due to its natural resistance to fluconazole, which can facilitate the acquisition of resistance to other antifungal drugs.^24^ Previously, three cases of rapid acquired resistance of echinocandins, such as micafungin and caspofungin, have been reported in a bone marrow transplant unit, with death outcome in one case.^25^ Previous in vitro tests, *Pichia kudriavzevii* presented, in 10 days of caspofungin exposure, alterations in FKS1 gene expression, which is related to decreased susceptibility to echinocandins and, consequently, treatment failure.^26^

The risk factors for invasive infection caused by *Pichia kudriavzevii* include prophylaxis with azoles, continuous antibiotic therapy, blood cancer, neutropenia and long hospitalization.^27^ *Pichia kudriavzevii* infection outbreaks have been reported, associated with prolonged use of carbapenems and disruption of the tissue barrier, such as the use of venous catheters and multiple medical devices.^28^ As demonstrated in some case reports, cases of pneumonia or severe *Pichia kudriavzevii* infections have been identified in patients with immunosuppression, such as transplant recipients or extremely low birth weight neonates.^29,30^ This highlights the critical need for the development of improved therapeutic strategies to combat this challenging pathogen, which has been described as a moderate priority by the WHO fungal priority pathogen list (FPPL), requiring better prevention, treatment and identification measures to avoid outbreaks and harm to public health worldwide.^31^ Despite advances in antifungal drugs, existing therapies are limited by toxicity and the rise of drug-resistant fungal species.

Antimicrobial photodynamic therapy (aPDT) is an alternative method for treating infections caused by fungi or bacteria, including resistant strains.^32^ aPDT involves the use of a photosensitizer in combination with visible light and oxygen in the ground state (O_2_(^3^S_g_)), causing photogeneration (via photosensitized reaction) of short-living, highly cytotoxic singlet oxygen (O_2_(^1^Δ_g_), and other reactive oxygen species (ROS), in near surrounding or inside microbial cells. ROS can induce chain reactions of damage to cellular structures such as proteins, nucleic acids and lipids, resulting in cell death.^33^ Resistance mechanisms of aPDT are rarely reported, due to its broad spectrum of action, and its low mutagenic potential in selecting resistant strains, which may contribute to the current scenario of multiresistant species.^34–36^ The “resistance problem”, including the resistance of structures such as biofilms, can be overcome by alternative therapies that utilize ROS. Biofilms can contain several sites for ROS action, such as extracellular DNA, lipids and proteins, which are essential for their stability and structure.^35^ The aPDT also allows application to biotic or abiotic surfaces, without harming the host cells, allowing the treatment and prevention of biofilms on tissues or even medical devices, which are subject to contamination.^37^ Several photosensitizers, such as porphyrins, toluidine/methylene blue or curcumin, have demonstrated antimicrobial effects on *Candida* species, including their biofilms in different development stages, demonstrating their antimicrobial potential against these pathogenic yeasts, increasing the arsenal of antifungal therapies currently available.^38–42^ In comparison with classical antifungal drugs, the antifungal effect of photosensitizers is limited only to the area irradiated by light.

Currently, the interaction between photosensitizers and nanoparticles (NPs) in aPDT has been shown. NPs offer significant advantages in overcoming the intrinsic limitations of drug delivery systems, significantly expanding its therapeutic potential. Nanoparticle-based systems permit controlled and sustained drug release, resulting in reduced dosing frequency and diminished systemic and local adverse effects.^43^ A variety of materials have been used to generate nanoparticles, and many photosensitizers have been combined or encapsulated to enhance their antimicrobial effects.^44^ NPs can be used to prevent the aggregation of some photosensitizers in aqueous solutions, especially porphyrin-derived photosensitizers, increasing their useful life and, consequently, their antimicrobial activity.^45^ Sulfonated polystyrene nanoparticles (NPs) are biocompatible, permeable to oxygen and transparent to visible light, permitting their use in aPDT.^46^ In comparison with individual porphyrin molecules, NPs with encapsulated porphyrin (TPP-NPs) photogenerated a higher local concentration of O_2_(^1^Δ_g_) and serve as a storage container, where O_2_(^1^Δ_g_) is steeply released from the NPs interior to the environment to attack biological targets.^31^ In contrast to free photosensitizers, excited states of TPP photosensitizer in oxygen permeable NPs are efficiently protected against quenching not only from aggregation but also from external molecules, except the O_2_(^3^S_g_) forming O_2_(^1^D_g_) via energy transfer. Additionally, TPP molecules encapsulated in NPs are not in direct contact with the environment, excluding any additional direct toxic effects. The light-induced antifungal activity of nanoparticles with an encapsulated porphyrin (TPP-NPs) was recently demonstrated in six *Candida* species, including *Candida albicans* and *Pichia kudriavzevii* (*Candida krusei*).^47,48^ Curiously, *Pichia kudriavzevii* was the most sensitive species to aPDT when TPP-NPs were used. In addition, potassium iodide (KI) was shown to potentiate the aPDT effect when TPP-NPs were used, resulting in a dual effect due to the photogeneration of O_2_(^1^Δ_g_) and the oxidation of I^–^ by O_2_(^1^Δ_g_) to I_2_/I_3_^–^ to create a create “in situ” an additional antimicrobial longer-lived species with longer cytotoxic range. It is suggested that the photoinactivation of these yeasts biofilms is quite promising, through the association of TPP-NPs with KI, with emphasis on *Pichia kudriavzevii*, considering its history of resistance to conventional antifungals.^24–26^

Therefore, the primary aim of this study was to evaluate the effectiveness of aPDT treatment mediated by TPP-NPs and the synergistic effect of potassium iodide on the formation and elimination of biofilms produced by two fungal species, namely *Candida albicans* and *Pichia kudriavzevii*, comparing the action of the treatment between the two species, and figure out how the treatment works.

## 2 Materials and Methods

### 2.1 Yeast Strains and Growth Conditions

*Candida albicans* (Manassas, VA, USA; ATCC #10231) and *Pichia kudriavzevii* (Manassas, VA, USA; ATCC #6258) were plated on Sabouraud dextrose agar (Merck, Darmstadt, Hesse, Germany) and incubated at 37 °C for 48 h in atmosphere air. After this period, colonies in the stationary growth phase were collected from the agar surface and suspended in sterile physiological saline (0.9% NaCl) to obtain a cell suspension. The suspension was then adjusted to a final density of 1 × 10^7^ cells·mL^–1^ by direct microscopic counting using a Neubauer chamber, considering total cells per milliliter. Cell counting was performed and the average number of cells per quadrant was used to calculate the final concentration. To prepare the cell solution at a concentration of 1 × 10^6^ cells. mL^–1^, the solution created previously was diluted in saline solution (0.9%) 10 times.

### 2.2 Preparation and Characterization of Stable Sulfonated Polystyrene Nanoparticles with Encapsulated Porphyrin (TPP-NPs)

5,10,15,20-Tetraphenylporphyrin encapsulated in sulfonated polystyrene nanoparticles (TPP-NPs) was synthesized via the nanoprecipitation method and characterized according to Kubát et al. (2017).^49^ The particle size, particle size distribution and zeta potential in water were determined by dynamic light scattering (DLS) on a Zetasizer Nano ZS particle size analyzer from Malvern. The generation of O_2_(^1^Δ_g_) was verified by measurements of its near-infrared luminescence.^50^ The ability of TPP-NPs to photo-oxidize iodide in aqueous media was assessed using the iodide method.^51,52^ Aqueous dispersions of TPP-NPs generating O_2_(^1^Δ_g_) with or without chemical (KI) and/or biological (yeast) targets were activated by visible light from an LED lamp (λ = 414 nm). The light source was placed 30 cm from the irradiated samples. The spectral irradiance of the LED lamp (Figure 1d) was recorded by an ILT960 Spectroradiometer SpectriLight (International Light Technologies). The properties of the TPP-NPs were preserved by storing them as a stock dispersion in the dark at room temperature.

**Figure 1:**
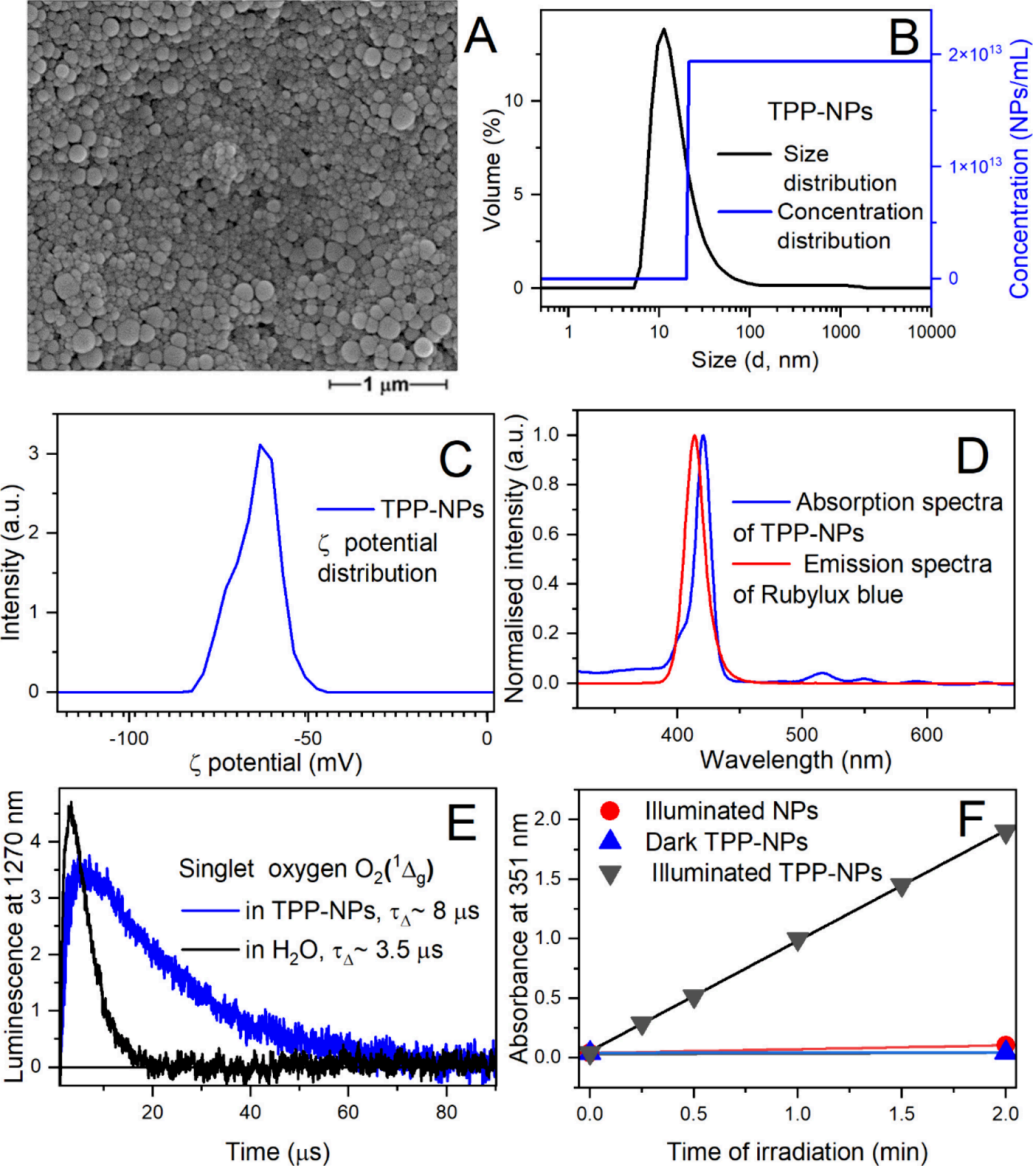
(A) The shape of TPP-NPs on the surface of the nanofiber membrane observed via SEM. (B) Size distribution of TPP-NPs and dependence of their concentration as a function of size in the stock dispersion. (C) Zeta potential of TPP-NPs. D: Absorption spectrum of TPP-NPs in H_2_O and the emission spectrum of the LED light used for their excitation. (E) Kinetics of singlet oxygen deactivation in water after irradiation of TMPyP (water-soluble photosensitizer) or after irradiation of a dispersion of TPP-NPs by a short laser pulse (∼7 ns, 355 nm). (F) Demonstration of the ability of TPP-NPs to photo-oxidize I^–^ to I_3_^–^, tested using the iodide method.

### 2.3 Effect of aPDT with TPP-NPs on Either *Candida albicans* or *Pichia kudriavzevii* growth

Cell suspensions (10^6^ cells·mL^–1^) (25 μL) were prepared according to section 2.1 and seeded in a 96-well polystyrene plate containing different concentrations of TPP-NPs (from 1 × 10^13^ to 6 × 10^8^ TPP-NPs·mL^–1^, prepared by serial dilution) either in the absence or presence of KI (10 mM) in a final volume of 200 μL. The cells were incubated in the dark for 5 min, after which the cover of the plate was removed, and the plates were irradiated with appropriate light at room temperature. Cells incubated only in sterile physiological medium were included as a control. The light source used was a light-emitting diode (Rubylux blue LED lamp) with an output power of 18 W and a peak wavelength of 415 nm positioned 10 cm from the irradiated plate. The laser beam illuminated an area of 176.71 cm^2^, and the 96-well polystyrene plate was positioned in this area and irradiated for 10 min. An increase of 5 °C in the medium temperature was observed during the irradiation of the samples. Importantly, this could be an advantage, since, as previously demonstrated, a slight increase in temperature increases the oxygen permeability of polystyrene NPs and therefore increases the kinetics of iodide photooxidation and antimicrobial effects (Kubát et al., 2019). Following irradiation, the cells remained in the dark (37 °C), and 25-μL aliquots were removed, after homogenizing the contents of the wells immediately, 20, or 50 min after irradiation. These aliquots were plated in 24-well plates containing Sabouraud dextrose-broth (SDB) medium (Merck, Darmstadt, Hesse, Germany) at a final volume of 1,000 μL. After 18 h of incubation at 37 °C, the contents of the wells were homogenized, and cell growth was determined by quantifying the optical density at 570 nm (OD_570_) using a Synergy HT Multi-Detection Microplate Reader. The experiments were performed under aseptic conditions. The data obtained are expressed as a percentage (%) of the growth relative to the control group (cells not irradiated and in the absence of TPP-NPs).

### 2.4 Effect of aPDT on Biofilm Formation by *Candida albicans* or *Pichia kudriavzevii*

*Candida albicans* or *Pichia kudriavzevii* suspensions (10^7^ cells·mL^–1^) were subjected to aPDT using TPP-NPs, according to the methodology presented above. Cells incubated only in sterile physiological medium were included as a control. At 20 and 50 min after irradiation (the medium was maintained in the dark), the medium was homogenized, and aliquots of 25 μL were taken and plated in 96-well plates containing either RPMI-1640 medium (Sigma, St. Louis, MO, USA) or SDB in a final volume of 200 μL. *Candida albicans* biofilms were produced using RPMI-1640 medium, and *Pichia kudriavzevii* biofilms were formed using SDB. The plates were incubated for 24 h at 37 °C to form biofilms. After this period, the medium was carefully removed, each well was washed 2 times with 200 μL of PBS to remove nonadherent cells, and 200 μL of PBS was added to each well. The metabolic activity of the biofilms was quantified using a metabolic assay based on the reduction of XTT (2,3-bis­(2-methoxy-4-nitro-5-sulfophenyl)-2H-tetrazolium-5-carboxanilide sodium salt) (Molecular Probes, Eugene, OR, USA). Prior to each assay, XTT solution (1 mg·mL^–1^) was thawed and mixed with a freshly prepared 0.4 mM menadione solution (a respiratory electron chain-uncoupling agent that accelerates respiration and XTT reduction; Sigma, St. Louis, MO, USA) at a volume ratio of 9:1. An aliquot of 20 μL from this mixture was added to each well. After 2 h, the colored reduced formazan product was measured at 490 nm (OD_490_) using a Synergy HT multiple-detection microplate reader. The experiments were performed under aseptic conditions. The results are presented as percentages (%), calculated relative to the metabolism of control group (cells not irradiated and in the absence of TPP-NPs) biofilms. The morphology of the biofilms was observed via light microscopy before the addition of XTT solution.

### 2.5 Effect of aPDT on the Metabolic Activity of 24-h Biofilms Produced by Either *Candida albicans* or *Pichia kudriavzevii*

Cellular suspensions (10^7^ cells·mL^–1^) (25 μL) were plated in 96-well plates containing RPMI-1640 medium (*Candida albicans*) or SDB (*Pichia kudriavzevii)* in a final volume of 200 μL. The cells were maintained for 24 h (37 °C) to produce biofilms. After biofilm formation, the medium was carefully removed, and each well was washed 2 times with 200 μL of PBS and incubated in the dark for 5 min at room temperature in the presence of 1 × 10^13^ TPP-NPs·mL^–1^ either with or without KI (10 mM) in a final volume of 200 μL. Cells incubated only in sterile physiological medium were included as a control. The cover of the plate was subsequently removed, and the plates were irradiated with appropriate light at room temperature. Immediately, 20, or 50 min after irradiation (the plates were kept in the dark), the reaction medium was removed, and each well was washed 2 times with 200 μL of PBS to remove nonadhered cells. The metabolic activity of the biofilms was quantified via the XTT assay according to the methodology described above. The results are presented as percentages (%), calculated relative to the metabolism of control group (cells not irradiated and in the absence of TPP-NPs) biofilms.

### 2.6 Morphological Analyses of Biofilms

The morphology of the biofilms produced by *Candida albicans* or *Pichia kudriavzevii* was evaluated using light microscopy (Axioskop 2, Zeiss, Jena, Germany). The images were captured on a Pixera digital camera system (Pixera Corporation, Santa Clara, CA, USA) coupled to the photomicroscope and a microcomputer (IntelVR PentiumVR, Santa Clara, CA, USA) using Adobe Photoshop version 7.0.1 (Adobe Systems, Atlanta, GA, USA).

### 2.7 Effect of aPDT Using TPP-NPs on ROS Production by Either *Candida albicans* or *Pichia kudriavzevii*

Cellular suspensions (10^6^ cells·mL^–1^) (25 μL) were seeded in a 96-well polystyrene plate containing TPP-NPs (1 × 10^13^ TPP-NPs·mL^–1^) either in the absence or presence of KI (10 mM) in a final volume of 200 μL. The cells were incubated in the dark for 5 min, after which the cover of the plate was removed, and the plates were irradiated with appropriate light at room temperature according to the protocol described in Section 2.3. Cells incubated only in sterile physiological medium were included as a control. Following irradiation, the cells remained in the dark (37 °C) until the contents of the wells were homogenized, and aliquots of 50 μL were taken (immediately, 20 and 50 min after irradiation) and plated in dark 96-well plates containing 2’,7’ dichlorodihydrofluorescein diacetate (H_2_DCF-DA) (0.050 μM) (Molecular Probes, Eugene, OR, USA) in PBS at a final volume of 200 μL. The plate was incubated for 1 h in the dark at 37 °C. After this period, the fluorescence intensity was determined using a Synergy HT multiple-detection microplate reader (Bio-Tek, Winooski, VT, USA) with excitation at 480 nm and emission at 530 nm. The experiments were performed under aseptic conditions. The data obtained are expressed in arbitrary units.

### 2.8 Statistical Analysis

The values are expressed as the means ± standard deviations (SD) of independent experiments (*n* = 8). Each independent experiment was performed in triplicate. The statistical technique used was one-way analysis of variance (ANOVA) followed by the Tukey–Kramer post hoc test for multiple comparisons. *P* values <0.05 were considered significant. Graphics were generated and statistical analysis was performed using OriginPro 8.5 (OriginLab Corporation, Northampton, MA, USA).

## 3 Results

### 3.1 Characterization of TPP-NPs

Initially, sulfonated polystyrene nanoparticles (NPs) with encapsulated hydrophobic tetraphenylporphyrin (TPP) photosensitizer (TPP-NPs) synthesized according to KUBÁT et al. (2017)^35^ were characterized. The prepared nanoparticle dispersions of both the NPs and TPP-NPs were mostly spherical (Figure 1A), with an average diameter of approximately 89 nm; however, most of the NPs and TPP-NPs were 10–20 nm in size, as shown in Figure 1B. The concentration of the NPs and TPP-NPs in the stock dispersions was 1.8 × 10^13^ NPs·mL^–1^ (Figure 1B). Most likely, the large average diameter is due to the existence of larger nanoparticles and the methodology used for their calculation. We found a relatively high ζ potential of approximately −76 mV (Figure 1C), which provides high stability of dispersion over time without aggregation. The absorption spectrum of TPP-NPs fits well with the emission spectra of the LED light source (λ_em_ = 414 nm) used for excitation (Figure 1D). The ability of TPP-NPs to photogenerate O_2_(^1^Δ_g_) was assessed by measuring its characteristic luminescence at 1270 nm (Figure 1E). The measured kinetics of the luminescence showed that a water-soluble photosensitizer photogenerated O_2_(^1^Δ_g_) in H_2_O, with a typically short lifetime of approximately 3.5 μs, whereas the lifetime of O_2_(^1^Δ_g_) in an aqueous dispersion of TPP-NPs was prolonged to approximately 8 μs. Because hydrophobic TPP is encapsulated inside the NPs, this value is a combination of the lifetime of O_2_(^1^Δ_g_) in NPs (less quenching environment) and in H_2_O (highly quenching environment). The ability of O_2_(^1^Δ_g_) to escape NPs and oxidize I^–^ (an external substrate) to I_3_^–^ in the aqueous environment was checked using the iodide method^52^ (Figure 1F).

### 3.2 Effects of aPDT on the Development and Viability of *Candida albicans* or *Pichia kudriavzevii* Biofilms

The effects of different concentrations of TPP-NPs on *Candida albicans* growth were determined (Figure 2). Cells subjected to aPDT with TPP-NPs (1 × 10^13^ TPP-NPs·mL^–1^) presented a growth reduction of 25%, whereas cells incubated in the presence of TPP-NPs without irradiation were not significantly affected (Figure 2A). When the cells were maintained for 50 min after aPDT, before removal and growth, a modest but consistent increase in growth inhibition was observed. Figure 2B shows the effect of aPDT using different concentrations of TPP-NPs in the presence of KI (10 mM). KI markedly potentiated the aPDT effect. With KI, a TPP-NP concentration 10^4^ times lower (3 × 10^9^ TPP-NPs·mL^–1^) produced the same effect observed in the absence of KI (25% inhibition), and 1 × 10^10^ TPP-NPs·mL^–1^ inhibited ∼99% of *Candida albicans* growth.

**Figure 2:**
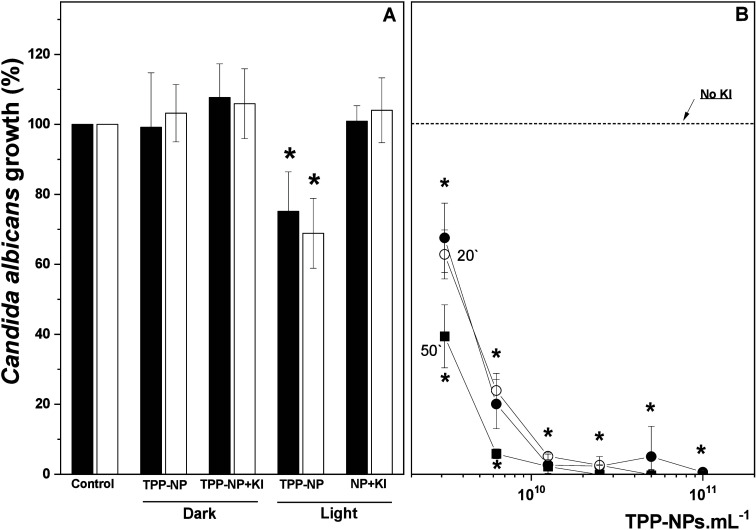
Effect of aPDT with TPP-NPs on *Candida albicans* growth. The experimental conditions are described in the Materials and Methods. (A) Cell suspensions were incubated under different conditions either in the dark or under irradiation. After different treatments, the cells were taken immediately (dark columns) or 20 min later (white columns) and incubated for 18 h. (B) Cell suspensions were treated with aPDT in the presence of KI and taken immediately (○), 20 (●), or 50 min (■) after irradiation. After 18 h of incubation, cell growth was determined by quantifying the optical density at 570 nm (OD_570_). The dotted line represents 100% of growth in the absence of KI. The data are shown as the means ± SD from eight independent experiments executed in triplicate and are expressed as the percentage (%) of growth relative to the control group (100%) (cells not irradiated and in the absence of TPP-NPs). * *p* < 0.05.

Furthermore, cells maintained for an additional 50 min after aPDT in the presence of KI presented greater growth inhibition. No effect was observed on cells irradiated in the presence of both NPs and KI or incubated with both TPP-NPs and KI but not irradiated (Figure 2A). Figure 3A shows the marked inhibitory effect of aPDT using TPP-NPs on *Pichia kudriavzevii* growth. A concentration of 8 × 10^10^ TPP-NPs·mL^–1^ produced 50% inhibition, although this same concentration did not affect *Candida albicans* growth (data not shown). aPDT using TPP-NPs (1 × 10^12^ TPP-NPs·mL^–1^) was able to reduce cell growth by 90–95%. This inhibition profile was not modified in the remaining cells after aPDT. As observed for *Candida albicans*, the inhibition of *Pichia kudriavzevii* growth by aPDT was more pronounced in the presence of KI (Figure 3B).

**Figure 3:**
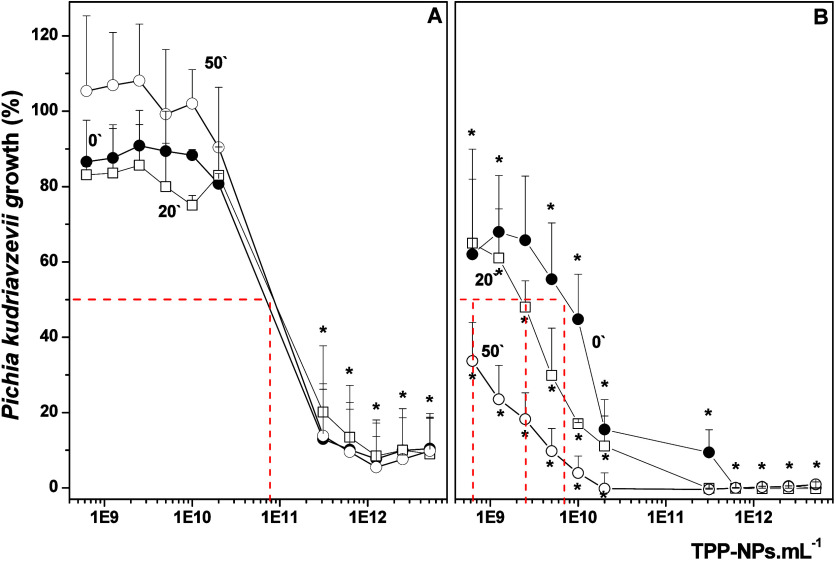
Effect of aPDT with TPP-NPs on *Pichia kudriavzevii* growth. The experimental conditions are described in the Materials and Methods. The cell suspensions were incubated with different concentrations of TPP-NPs and irradiated either in the absence (A) or presence (B) of 10 mM KI. After aPDT, the cells were taken immediately (●), 20 (□), or 50 min (○) after irradiation and incubated for 18 h, after which the cell growth was determined by quantifying the optical density at 570 nm (OD_570_). The data are shown as the means ± SD from eight independent experiments executed in triplicate and are expressed as the percentage (%) of growth relative to the control group (100%) (cells not irradiated and in the absence of TPP-NPs). *p < 0.05.

The TPP-NP concentration necessary to reduce cell growth by 50% was 8 × 10^10^ TPP-NPs·mL^–1^ in the absence of KI and 8 × 10^9^ TPP-NPs·mL^–1^ in the presence of KI, indicating a reduced ability of KI to potentiate the effects of aPDT on *Pichia kudriavzevii* compared to its potentiation capacity against *Candida albicans*. The growth of cells allowed to rest after aPDT in the presence of KI was decreased compared to cells collected immediately following irradiation, suggesting continued effects. These results demonstrated the ability of aPDT using TPP-NPs to decrease both *Candida albicans* and *Pichia kudriavzevii* growth. Because the ability to produce biofilms is a crucial step in colonization and infection by these microorganisms, the effect of aPDT using TPP-NPs on biofilm formation was determined. Figure 4 shows the inhibition of biofilm formation by aPDT for *Candida albicans* and *Pichia kudriavzevii.* A 15, 37, and 50% reduction in biofilm formation was observed in *Candida albicans* cells treated with aPDT and collected immediately, 20, or 50 min after aPDT, respectively (Figure 4A). The addition of KI during aPDT with TPP-NPs enhanced the inhibitory effect; ∼97% inhibition of biofilm formation was observed. A very similar inhibition profile was observed for biofilm formation by *Pichia kudriavzevii* treated with aPDT using TPP-NPs, both in the presence and in the absence of KI (Figure 4B).

**Figure 4:**
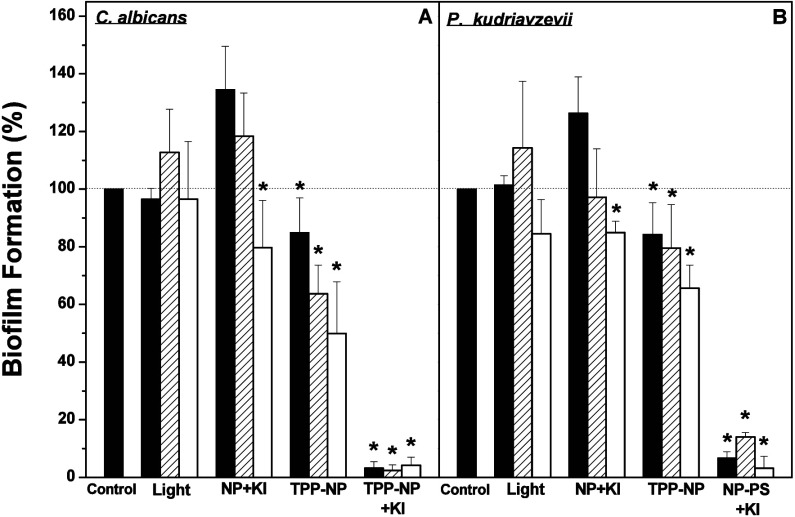
Effect of aPDT involving the use of TPP-NPs on biofilm formation by *Candida albicans* (A) or *Pichia kudriavzevii* (B). The experimental conditions are described in the Materials and Methods. *Candida albicans* or *Pichia kudriavzevii* suspensions were incubated under different conditions either in the dark or under irradiation. The cells incubated only in sterile physiological medium were included as the control. After different treatments, the cells were taken immediately (dark columns), 20 (striped columns), or 50 min later (white columns) and incubated for 24 h to form biofilms. After this period, the metabolic activity of the biofilms was determined using XTT assays. The values presented in the figure represent the percentage of the metabolic activity of control biofilms, calculated using the control group (cells not irradiated and in the absence of TPP-NPs) biofilms as 100%. The data are presented as the means ± SD (*n* = 8). *p < 0.05.

The inhibitory effect was also observed by analyzing the morphology of cells present in biofilms using light microscopy (Figure 5).

**Figure 5:**
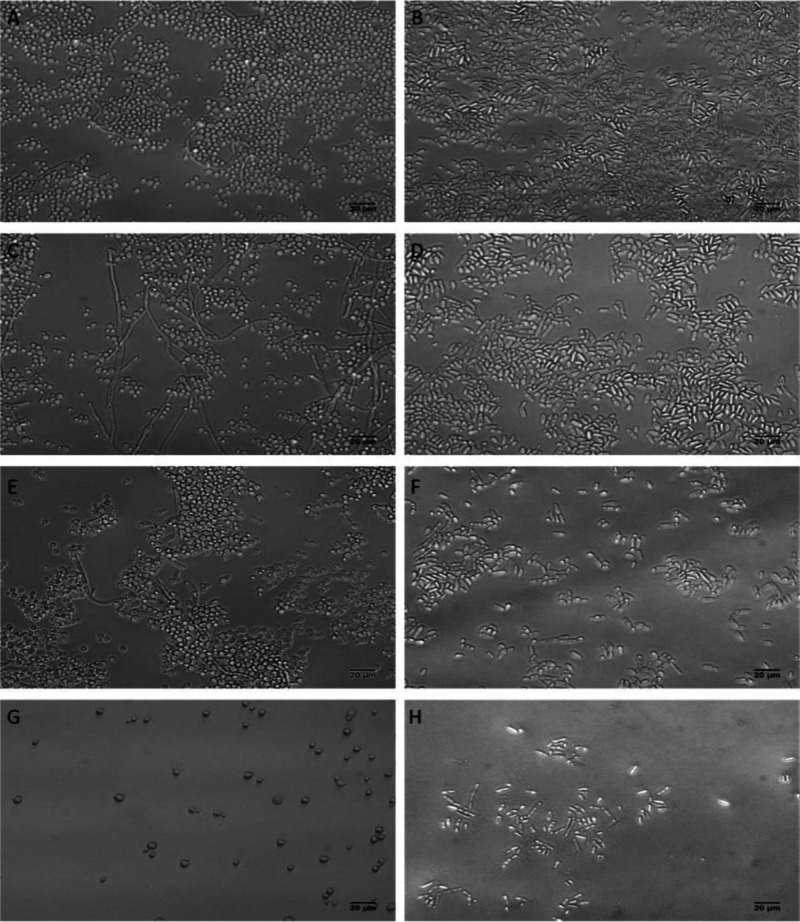
Morphological characterization of biofilm formation by *Candida albicans* (A, C, E, G) and *Pichia kudriavzevii* (B, D, F, H). Cell suspensions not treated (control group) (A, B), irradiated in the absence of TPP-NPs (C, D), or treated with aPDT using TPP-NPs (1 × 10^13^ TPP-NPs·mL^–1^) either in the absence (E, F) or in the presence (G, H) of 10 mM KI were incubated for 24 h to form biofilms.

Compared with the control group (Figure 5A and 5B), a reduction in the number of cells present in the biofilm structure was observed for both *Candida albicans* and *Pichia kudriavzevii* (Figure 5E and 5F) when the cells were treated with aPDT. The presence of a mixture of yeast cells and filaments in the biofilm structure of the control group and the presence of either spherical or cylindrical yeast cells, characteristic of *Candida albicans* and *Pichia kudriavzevii,* respectively, were observed (Figures 5A and 5B). The addition of KI during aPDT promoted a great reduction in the number of cells present in the biofilm, and a total absence of cells presenting filamentous forms was noted (compare Figures 5A and 5B with Figures 5G and 5H). Cells that only experienced irradiation (Figures 5C and 5D) or that were incubated with TPP-NPs but not irradiated (data not shown) did not display significantly altered biofilm formation. In addition, control group, cells with just irradiation and with TPP-NPs irradiated (Figures 5A, 5C and 5E) shown germ tubes in *Candida albicans* biofilm, the presence of KI associated with TPP-NPs (Figure 5G) denotes the absence of these elongated structures. Taken together, these results demonstrated the potential of aPDT using TPP-NPs, mainly in the presence of KI, to decrease the development of both *Candida albicans* and *Pichia kudriavzevii.* The cells present in the structure of mature biofilms are several times more resistant to antifungal therapy than planktonic cells, making the eradication of these structures a challenge worth pursuing. A 30–40% reduction in the metabolic activity of 24-h biofilms treated with aPDT and TPP-NPs was observed with *Candida albicans* (Figure 6A).

**Figure 6:**
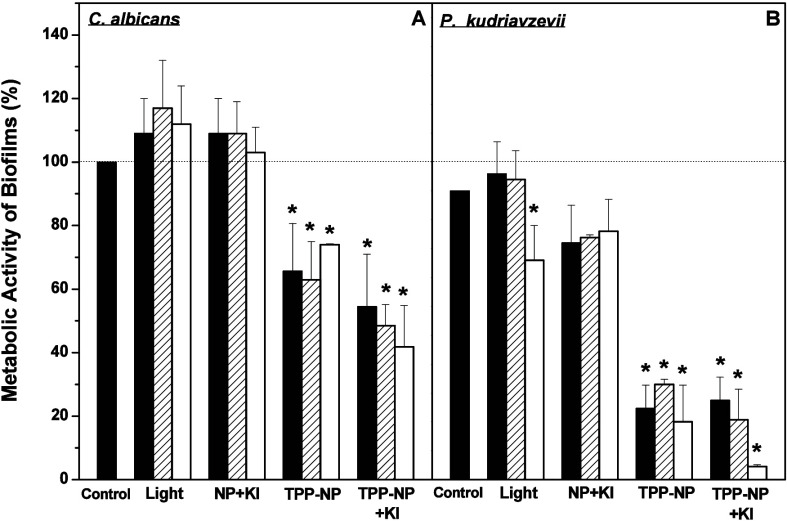
Effect of aPDT using TPP-NPs on the metabolic activity of 24-h biofilms produced by *Candida albicans* (A) or *Pichia kudriavzevii* (B). Details of the experimental conditions are described in the Methods section. A cell suspension was used to produce biofilms for 24 h. After this period, 24-h biofilms were treated with aPDT (1 × 10^13^ TPP-NPs·mL^–1^), either in the absence or presence of KI (10 mM). Biofilms incubated only in sterile physiological solution were included as the control. The metabolic activity of the biofilms formed was quantified using XTT assays immediately (dark columns), 20 (striped columns), or 50 min (white columns) after aPDT. The values presented in the figure represent the percentage of the metabolic activity of control biofilms, calculated using the control group (cells not irradiated and in the absence of TPP-NPs) as 100%. The data are presented as the means ± SD (*n* = 8). * *p* < 0.05.

The presence of KI increased the metabolic activity inhibition by aPDT to 50–60%. Furthermore, the metabolic activity of 24-h biofilms produced by *Pichia kudriavzevii* decreased 70–80% after treatment with aPDT using TPP-NPs in the presence of KI, and this effect was potentiated after 50 min (80–95% inhibition) (Figure 6B). The metabolic activity of biofilms either only irradiated or incubated in the presence of NP or KI was not modified for either *Candida albicans* or *Pichia kudriavzevii*; however, in *Pichia kudriavzevii*, decreased metabolic activity was observed in biofilms maintained for 50 min after only irradiation. Mature biofilms produced by *Candida albicans* (24 h) present a complex structure with cells in both planktonic and filamentous forms, and many long filaments and structures related to invasion and colonization by the fungus can be observed (Figure 7A).

**Figure 7:**
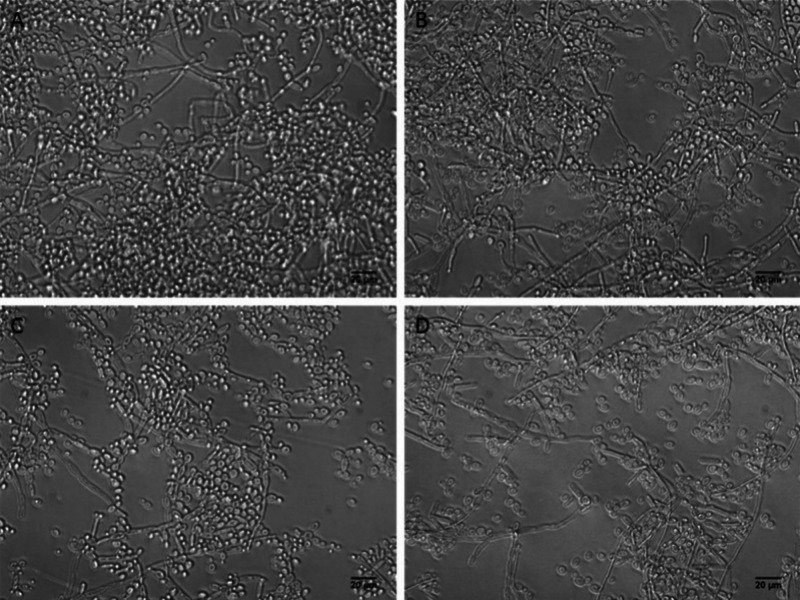
Morphological analyses of 24-h biofilms produced by *C. albicans*. Details of the experimental conditions are described in the Methodology section. Twenty-four hour biofilms were not treated (control group) (A), only irradiated in the absence of TPP-NPs (B), or treated with aPDT (1 × 10^13^ TPP-NPs·mL^–1^) either in the absence (C) or presence (D) of KI (10 mM).

Irradiation alone (in the absence of TPP-NPs) had no effect on biofilms (Figure 7B). However, 24-h biofilms treated with aPDT presented a reduction in the number of cells, both in planktonic and in filamentous forms present in the biofilm structure (Figure 7C). This reduction was greater in biofilms treated with aPDT in the presence of KI (Figure 7D). Furthermore, 24-h biofilms produced by *Pichia kudriavzevii* and treated with aPDT presented a major reduction in the number of cells present in biofilms, especially in the presence of KI (Figure 8).

**Figure 8:**
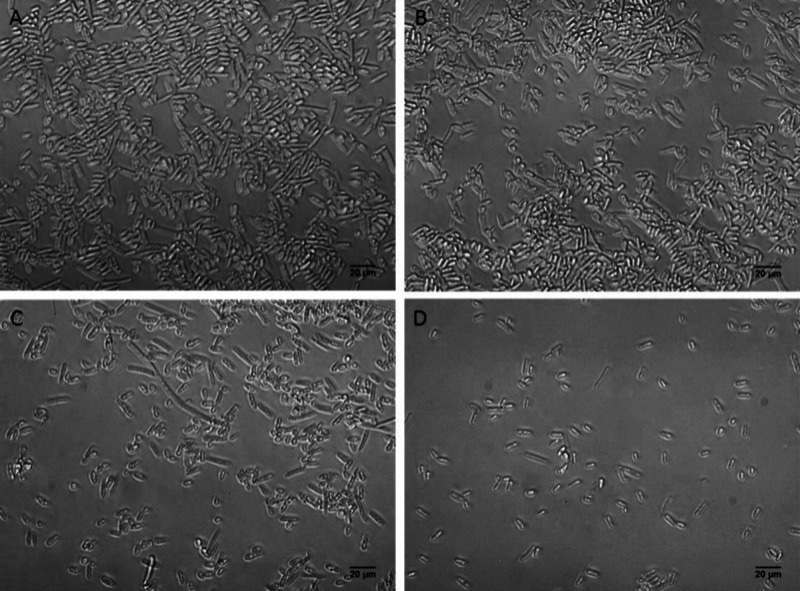
Morphological analyses of 24-h biofilms produced by *Pichia kudriavzevii*. Details of the experimental conditions are described in the Methodology section. Twenty-four hour biofilms were not treated (control group) (A), only irradiated in the absence of TPP-NPs (B), or treated with aPDT (1 × 10^13^ TPP-NPs·mL^–1^) either in the absence (C) or presence (D) of KI (10 mM).

A comparison of Figures 7 and 8 revealed that 24-h biofilms produced by *Pichia kudriavzevii* are much more sensitive to aPDT than biofilms produced by *Candida albicans*. These results suggest that photo-oxidation therapy could decrease the ability of yeast cells to adhere to the plate surface, demonstrating the potential of aPDT with TPP-NPs to eradicate cells present in biofilm structures, especially in biofilms produced by *Pichia kudriavzevii.* In addition, ROS production was determined both in *Candida albicans* and in *Pichia kudriavzevii* after aPDT (Table 1).

**Table 1:** ROS Measurement in *C. albicans* and *Pichia kudriavzevii* Cells Treated with aPDT Using TPP-NPs Either in the Presence or Absence of KI^a^

	*C. albicans*		*P. kudriavzevii*	
	Dark	aPDT	Dark	aPDT
Control	3.10 ± 2.38	3.83 ± 2.99	4.67 ± 1.15	5.00 ± 1.73
TPP-NPs	2.80 ± 1.99	5.33 ± 3.93	5.33 ± 1.53	6.00 ± 1.00
TPP-NPs + KI	3.25 ± 2.90	25.50 ± 4.04*	5.00 ± 1.00	31.67 ± 0.58*

a Details of the experimental conditions are described in the Materials and Methods section. The cell suspensions were incubated with TPP-NPs (1 × 10^13^ TPP-NPs·mL^–1^) either in the absence or in the presence of KI (10 mM) and irradiated. The cells incubated only in sterile physiological medium were included as the control. Immediately after irradiation, ROS production was determined using the H_2_DCF-DA assay. The values presented in the table represent the fluorescence intensity of the reaction expressed in arbitrary units. The experiments were performed under aseptic conditions. The data are presented as the means ± SD (*n* = 8). *p < 0.05.

Compared with the control group, no changes in ROS production were detected in the cells that were only irradiated, incubated in the presence of TPP-NPs in the dark, or subjected to aPDT in the absence of KI, either for *Candida albicans* or for *Pichia kudriavzevii.* However, an increase in ROS production was observed after aPDT with TPP-NPs in the presence of KI for both *Candida albicans* and *Pichia kudriavzevii*. These results demonstrated the potential of aPDT using TPP-NPs, especially in the presence of KI, to inhibit the development of both *Candida albicans* and *Pichia kudriavzevii.*

## 4 Discussion

In this work, we demonstrated the ability of aPDT using TPP-NPs to reduce the development of biofilms produced by either *Candida albicans* or *Pichia kudriavzevii*. Biofilms are recognized as significant virulence factors, and their presence on medical devices and host tissues is related to increased morbidity and mortality in hospitalized patients.^53,54^ Furthermore, the biofilm environment provides multiple benefits that increase the survival of the microorganisms residing within it,^55^ making biofilm-related infections especially difficult to eradicate. Although aPDT has proven effective in eliminating various microorganisms, including *Candida* species, compared with planktonic cells, the cells within biofilm structures show greater resistance to aPDT.^56^

The potential of aPDT using TPP-NPs as an antimicrobial therapy has been demonstrated against multiresistant pathogenic bacterial strains^48^ and, in combination with KI, against different *Candida* species.^47^ The results presented here revealed a reduction in cell proliferation after aPDT and a key ability of KI to increase the antifungal effect. This is important information, as the decrease in the concentration of TPP-NPs required to inhibit fungal development translates to a reduction in the dose administered. An important feature observed in this work is the greater sensitivity of *Pichia kudriavzevii* compared to *Candida albicans* when aPDT using TPP-NPs is applied. Despite being rare, *Pichia kudriavzevii* is a well-known pathogen due to its intrinsic resistance to fluconazole and reduced susceptibility to amphotericin B, producing a high mortality rate (49%).^27,57,58^ For this reason, new therapies with the potential to inhibit *Pichia kudriavzevii* development are crucial.

In this study, we used an improved aPDT method based on NP-TPPs in the presence of a low concentration of nontoxic KI to generate two antimicrobial species, O_2_(^1^Δ_g_) and I_2_/I_3_^–^ (products of I^–^ oxidation by O_2_(^1^Δ_g_)), upon visible light excitation (Figure 9).

**Figure 9:**
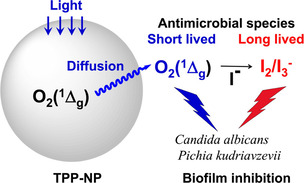
Simplified scheme of the antimicrobial effects of TPP-NPs with or without KI on *Candida albicans* and *Pichia kudriavzevii*.

Singlet oxygen (O_2_(^1^Δ_g_)) is formed by a photosensitization process after irradiation with a photosensitizer (TPP) inside polystyrene nanoparticles (with a lifetime of more than 8 μs) and diffuses easily into the environment. Because it has a short lifetime in aqueous solution (approximately 3.5 μs), O_2_(^1^Δ_g_) is able to photo-oxidize biological targets only near the TPP-NPs. However, the antimicrobial effect is amplified by the formation of more stable I_2_/I_3_^–^, which can hit biological targets at a greater distance from the surface of TPP-NPs. Curiously, the ability of KI to increase the inhibitory effect of aPDT using TPP-NPs was greater in *Candida albicans* than in *Pichia kudriavzevii*, suggesting different mechanisms of action. The mechanism by which KI potentiates aPDT using TPP-NPs has been related to a dual effect due to the photogeneration of O_2_(^1^Δ_g_) and I_2_/I_3_^–^, which serve as another antimicrobial species.^47^ Thus, the strong inhibitory effect of aPDT on *Pichia kudriavzevii* could be attributed to increased sensitivity to metabolites (including O_2_(^1^Δ_g_)) produced by aPDT using TPP-NPs, but the inhibitory effect of KI mediated I_2_/I_3_^–^ production would be lesser than that observed in *Candida albicans*. *Candida albicans* seems to be more sensitive to I_2_/I_3_^–^ produced during aPDT when TPP-NPs are used in the presence of KI. KI associated with photosensitizers can enhance the exchange of electrons and increase ROS production during aPDT.^59,60^

Previously, the effects of KI to enhance aPDT have already been attested by other authors, in microorganisms such as methicillin-resistant *Staphylococcus aureus*, including tests on cell lines such as HeLa, showing no toxicity. Still, any authors emphasized the inability of *Staphylococcus aureus* and *Salmonella paratyphi* to acquire resistance to aPDT associated with PS Rose Bengal associated with KI after numerous sessions of treatment.^61,62^ Pitaksanurat and collaborators (2024), achieved an effect similar to that of nystatin in *Candida albicans* by associating KI with PS erythrosine, through the generation of singlet oxygen.^63^ It is true that the effects of KI are beneficial and potentiating for aPDT, including in different biofilms, and depend on the capacity of singlet oxygen production by the PS and the cell membrane of the target microorganism for this action to be present.^59,64,65^ Added to this relevant information and the growing search for alternative methods for treating infections, the data from the present work contribute to elucidating and increasing the potential use of aPDT as an alternative. Interestingly, our results revealed an increase in ROS production after aPDT when TPP-NPs were used in the presence of KI, both in *Candida albicans* and in *Pichia kudriavzevii*, whereas in the absence of KI, aPDT did not affect ROS production. This result was surprising because an increase in ROS production after aPDT, even if a small increase, was expected in the absence of KI, especially in *Pichia kudriavzevii.*

Furthermore, the results presented in this work clearly demonstrated the antibiofilm effects of aPDT using TPP-NPs on *Candida albicans* and *Pichia kudriavzevii*. Atiencia-Carrera et al. (2022)^54^ reported a mortality rate of 37.9% in *Candida*-related bloodstream infections, whereas the mortality associated with biofilm-forming infections was reported to be 70% in a study analyzing the relationship between biofilms and mortality by infections associated with *Candida* spp. between 1995 and 2020. The results presented in this work demonstrated, for the first time, the ability of aPDT with TPP-NPs to reduce both biofilm formation and biofilm viability. *Candida* infections linked to biofilms can tolerate significantly higher concentrations of antifungal drugs than infections caused by planktonic cells, making biofilm-related infections particularly difficult to treat.^28^ In addition, the development of biofilms can lead to refractory infections and stimulate the development of other opportunistic infections.^11^ In the current research, different effects of the proposed treatment were achieved at different stages of the biofilm. When aPDT with TPP-NPs was applied early in *Candida albicans* biofilm formation (Figure 5C), we observed a reduction in germ tubes when compared to the control group (Figure 5A), with this effect being enhanced by KI (Figure 5E). Germ tubes are precursor structures of true hyphae and contribute to the structuring and invasion of the biofilm.^66^ Furthermore, the efficiency of aPDT in biofilm formation, using TPP-NPs with KI in *Candida albicans* was greater (97%) compared to the mature biofilm (30–40%), reinforcing the hypothesis that more mature biofilms, containing complex filaments, are challenging for any treatment, including aPDT. RPMI-1640 medium was used to form mature biofilms, which presented a greater number of filamentous (Figure 7). The medium and maturation time (>24-h), already present before treatment, are essential to hyphae formation, with RPMI-1640 medium providing nutrients necessary to induce the growth of these structures.^67^ The filaments present in *Candida albicans* biofilms ensure host tissue invasion, forming a scaffold embedded in the extracellular matrix, which is responsible for the robustness of the biofilm and, consequently, its greater difficulty in eradication. This complex structure increases the dispersal of new cells to other infection sites and allows nutrient exchange between fungal cells.^12,68^ These data are relevant to highlight the importance of identifying initial infections to prevent the formation and maturation of biofilms, in order to facilitate treatment. To compare the different *Candida* species, we observed 30–40% and 70–80% inhibition by aPDT in 24-h biofilms produced by *Candida albicans* and *Pichia kudriavzevii*, respectively. In *Pichia kudriavzevii*, the inhibition rate increased from 70–80% to 95% in 24-h biofilms treated with aPDT in the presence of KI. These two species present different challenges in terms of treatment and infection control. *Candida albicans* has a very complex and structured biofilm, which increases the chances of infections by multiple microorganisms and, consequently, morbidity and mortality.^69–71^ *Pichia kudriavzevii,* on the other hand, presents inherent difficulties with currently available treatments.^25,26^ *Candida albicans* proved more resistant to mature biofilm treatment, replicating what already occurs with other available treatments, but proved highly susceptible when treatment was applied early and associated with KI. However, *Pichia kudriavzevii* stood out, as it proved highly susceptible to aPDT with TPP-NPs, with or without KI, at all stages of the biofilm. This powerful inhibitory effect of aPDT using TPP-NPs on biofilms produced by *Pichia kudriavzevii* needs to be highlighted because of the known resistance of this microorganism to conventional antifungal therapies. Thus, aPDT with TPP-NPs could be an efficient and alternative antifungal therapy, especially considering the biocompatibility and high efficiency of the NPs.

## 5 Research Limitations

All research has limitations that can interfere with the data analysis and interpretation. The main limitation of this work was the experiments performed with *Pichia kudriavzevii*, since obtaining reproducible results in biofilm production by P. *kudriavzevii* is not easy. The production of robust biofilms, resistant to washing requires a great deal of attention and care from the researcher. Frequently, the biofilms detach from polystyrene plate during the experiment. Furthermore, other microscopy techniques would be enriching for evaluating biofilms in more depth, such as CLSM or/and SEM images.

## 6 Conclusion

This research demonstrates how aPDT impacts the development of biofilms and effectively diminishes or eliminates established biofilms, which are resistant to standard antifungal therapies. The findings could have significant implications for developing novel strategies to combat fungal biofilm-associated infections, considering the increasing resistance to standard antifungal agents. The study’s outcomes may contribute to optimizing aPDT protocols and exploring the synergistic effects of TPP-NPs and KI in clinical applications, potentially leading to more effective management of fungal biofilm-related diseases.
